# First case report of ensartinib in a patient with metastatic *ALK* rearranged lung cancer with *ALK* I1171N mutation: a case report

**DOI:** 10.1186/s12957-023-02935-9

**Published:** 2023-03-02

**Authors:** Shuang Yang, Weineng Feng, Yanming Deng, Jianmiao Liang

**Affiliations:** Department of Head, Neck and Thoracic Oncology, First People’s Hospital of Foshan, Foshan, China

**Keywords:** Ensartinib, *ALK* rearranged, *ALK* I1171N, Lung caner

## Abstract

**Background:**

The acquired resistance to *ALK* tyrosine kinase inhibitors (TKIs) in *ALK*-rearranged NSCLC is associated with poor survival outcomes and poses distinct clinical challenges. It is essential to develop potential therapeutic strategies for overcoming resistance.

**Case presentation:**

Here, we first report a female lung adenocarcinoma patient with an acquired *ALK* resistance mutation (*ALK* 11171N) who was treated with ensartinib. Her symptoms significantly improved after only 20 days, and with a side effect of mild rash. Follow-up images observed no further brain metastases after 3 months.

**Conclusions:**

This treatment may provide a new therapeutic strategy for *ALK* TKIs resistant patients, especially in position 1171 of *ALK* exon20.

## Introduction

Non-small cell lung cancer (NSCLC) remains the leading cause of death worldwide, with most patients being diagnosed at advanced stages of the disease. Alectinib is now one of the first-line treatment options for *ALK*-positive NSCLCs who have developed resistance to crizotinib, and most patients respond to alectinib with an objective response rate (ORR) of about 50% [1]. However, patients with *ALK*-positive inevitably relapse within a year or two of treatment due to various resistance mechanisms, of which *ALK* I1171N was found to be highly resistant to alectinib in previous reports [2]. In recent years, ensartinib has become a new first-line treatment option for patients with *ALK*-positive NSCLC, which shows potent inhibition of wild-type *ALK* and most common crizotinib-resistant mutations (such as F1174 and C1156Y, G1269A, L1196M, S1206R, and T1151) [3]. Notably, F1174 and C1156Y have acquired resistance mutations to second-generation *ALK* TKIs that have been clinically reported, suggesting that ensartinib may become a potential treatment for second-generation resistance to *ALK *[4]. In this case report, we report a lung adenocarcinomas patient harboring an acquired resistance mutation *ALK* I1171N who showed good responses with ensartinib.

## Case presentation

**Fig. 1:**
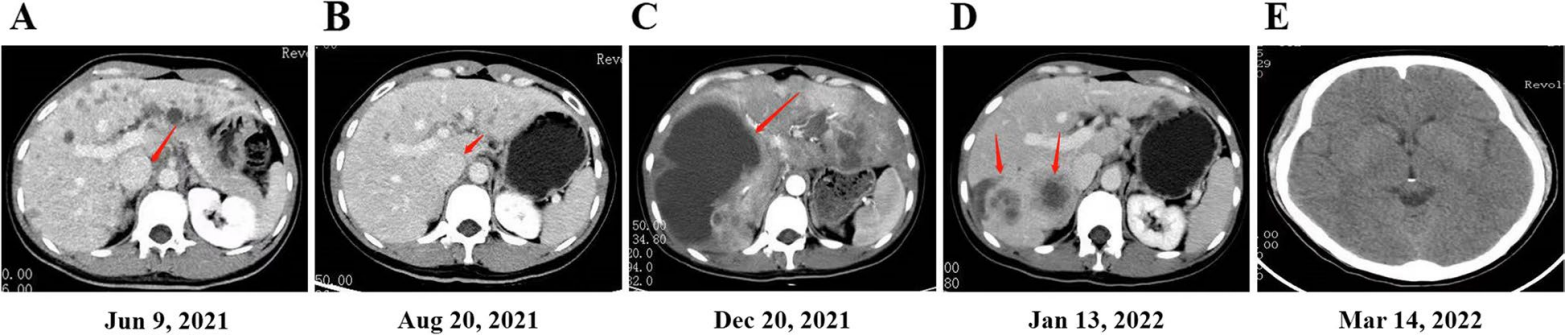
The patient’s treatment history and chronological imaging follow-up results. **A** CT of the abdomen before alectinib treatment (The red arrow indicates new metastases in the liver). **B** CT of the abdomen after 3 months of alectinib treatment (The red arrow indicates that the metastases in the liver were reduced). **C** CT of the abdomen before treatment of ensartinib treatment (The red arrow shows that the multiple liver lesions were significantly increased and enlarged). **D** CT of the abdomen after 20 days of ensartinib treatment (The red arrows show that the multiple liver lesions were significantly reduced). **E** Brain MRI after 3 months of ensartinib treatment

**Fig. 2:**
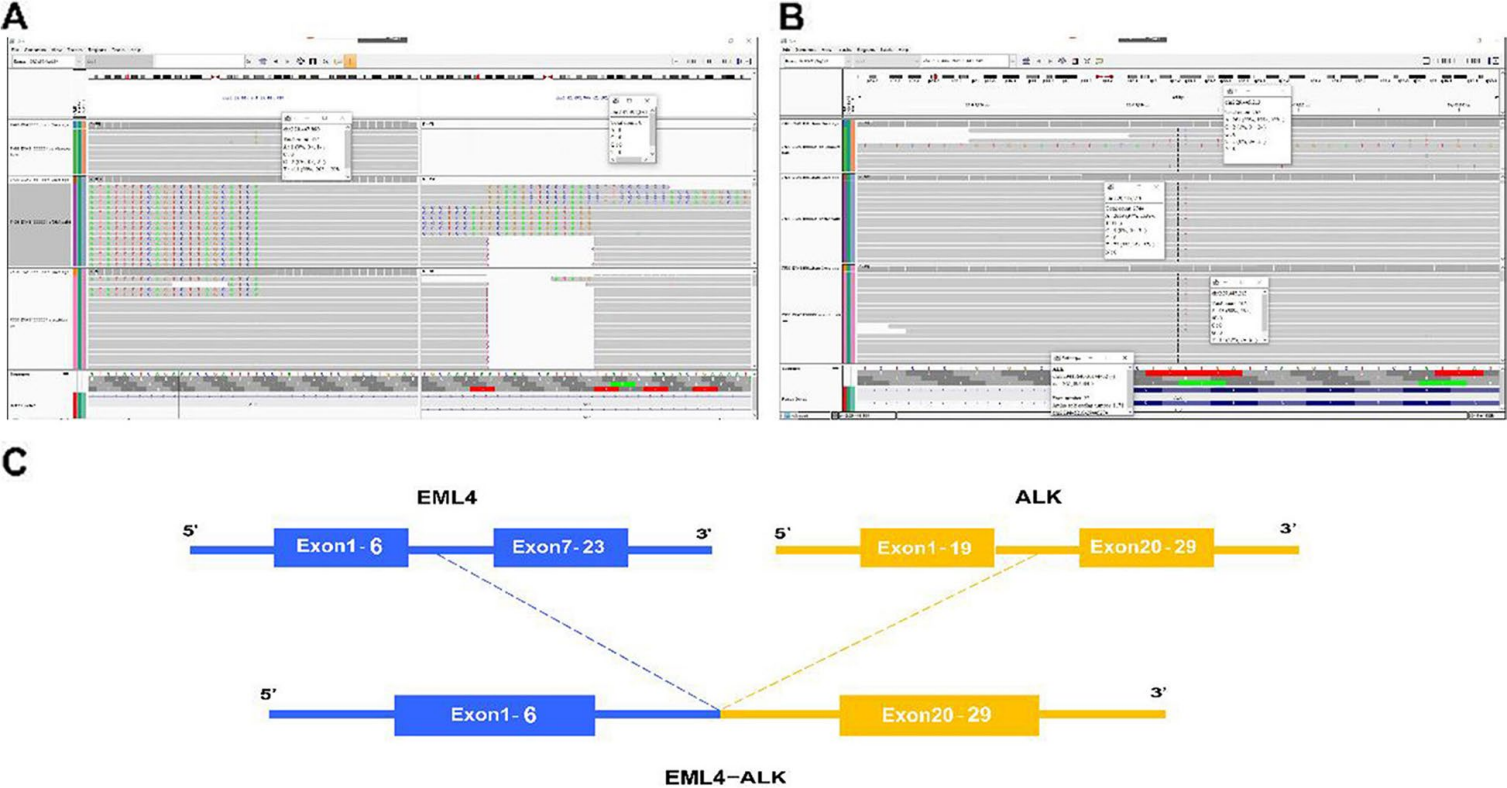
Detection of *EML4-ALK* fusion and *ALK* I1171N mutation. **A** The integrative genomics viewer screenshot of *EML4-ALK* fusion, detected by DNA-based NGS. **B** The integrative genomics viewer screenshot showed the *ALK* I1171N mutation. **C** Schematic representation of the *EML4-ALK* fusion structure

In March 2021, a 35-year-old female nonsmoker presented in another hospital with a lump located in her left neck. Ultrasound showed multiple abnormal lymph nodes in the left neck and suprasternal fossa. PET/CT showed a malignant lesion in the left posterior mediastinum and multiple metastases in the lungs, liver, and bones. A percutaneous biopsy on the left neck indicated a pathological diagnosis of T4N3M1c (IVB) metastatic lung adenocarcinoma. Next-generation sequence (NGS) analysis of the patient was performed to seek potential therapeutic regimens. This analysis revealed an *EML4*-*ALK* fusion (level, tissue 13.9%). The patient started oral alectinib 600 mg twice daily since May 2021. The abdominal CT images showed liver metastases accompanying multiple lymph node metastases in the hilar region one month later (Fig. 1A). The chest CT images still showed a malignant lesion in the left posterior mediastinum and diffuse bone metastases in the thoracolumbar spine. After 3 months of treatment, the chest and abdominal CT images showed thoracolumbar and bilateral multiple rib bone metastases were basically the same as before, multiple lymph node metastases in mediastinum, retroperitoneum and liver were less and smaller. According to the investigator’s assessment, the patient achieved a partial response at the first radiologic re-evaluation performed (Fig. 1B). However, her disease progressed after 7 months. Multiple liver lesions were observed to be significantly increased and enlarged than before (Fig. 1C). A percutaneous liver biopsy indicated adenocarcinoma. The liver metastases lesion and ctDNA were performed by NGS analysis and detected *EML4*-*ALK* fusion (level, plasma 0.24%) again, accompanied by an acquired *ALK* resistance mutation (*ALK* I1171N) (level, tissue 12.26%) (Fig. 2). During the period of taking alectinib, adverse event (AE) of the patient was increased alanine aminotransferase concentrations. Until December 2021, the patient stop took alectinib. According to the clinical treatment guidelines, the patient then took ensartinib as the second-line treatment immediately post alectinib. Surprisingly, 20 days later, CT re-examination showed that the multiple tiny nodules in both lungs were roughly the same as before, ultrasound re-examination revealed that most of the multiple lesions in the liver were reduced compared to before (Fig. 1D). AEs of the patient primarily included rash and pruritus (grade I), these symptoms significantly improved after allergy treatment. The patient did not appear to have noticeable brain metastases until March 14, 2022 (Fig. 1E).

## Discussion

Here we reported an *EML4*-*ALK* fusion NSCLC patient with an acquired resistance mutation *ALK* I1171N, who benefited from ensartinib. The patient’s disease progressed after a 7-month-treatment of alectinib, and *ALK* I1171N mutation, a resistance mutation, was detected in the liver metastases. After switching to ensartinib, most of multiple lesions in the liver were reduced. The patient’s condition is relatively stable so far, and we will continue to follow-up. To our knowledge, this is the first case reporting ensartinib as the second-line treatment for the *ALK* I1171N mutation. Ensartinib is active against a broad array of *ALK* mutations, including C1156Y, F1174, G1269A, I1171, L1152R/V, and even G1202R [4, 5], some of which are one of the resistance mechanisms after ceritinib or alectinib treatment [6]. Hence, for the patients who gained the above mutation sites after the treatment of ceritinib or alectinib, ensartinib can be used as a treatment option to overcome drug resistance. A review of studies on *ALK* inhibitors showed that half maximal inhibitory concentration (IC_50_) value of crizotinib on *ALK* activity was 4.5 nmol/L. In comparison, the IC_50_ value of ensartinib on *ALK* activity was < 0.4 nmol/L, which indicated that ensartinib has a stronger inhibitory effect [7]. In a phase II multicenter trial, 52% of patients with *ALK*-positive NSCLC had objective response (OR) after the treatment of ensartinib and the median progression-free survival (PFS) was 9.6 months [5]. A phase I/II multicenter study showed that ensartinib also has a strong inhibitory effect on *ALK* tyrosine kinase with specific mutation sites, including F1174 and C1156Y, and their IC_50_ value was both less than 0.4 nmol/L; the response rate (RR) was 60% and median PFS was 9.2 months among the *ALK*-positive evaluable patients, the median duration of response (DOR) for *ALK*-positive evaluable patients at data cut-off was 12.8 months [6]. Moreover, the ORR of patients with I1171 mutation was relatively lower than other *ALK* alterations [6, 8]. In addition, gastrointestinal (e.g., diarrhea and vomiting), ocular, and cardiac toxic effects were less frequent with ensartinib; therefore, these results indicated that ensartinib has relatively good safety [3]. Furthermore, ensartinib showed superior systemic, especially intracranial efficacy, which is essential considering that the central nervous system is a common disease progression [6]. Consistent with the above results, our case did not experience obvious adverse effects and developed brain metastases. However, further clinical trials are needed to validate the effect of ensartinib on *ALK* I1171N in advanced NSCLCs.

## Conclusions

In summary, our case report expands new treatment strategies for resistance to *ALK* TKIs, particularly the types of position 1171 of *ALK* exon20. Long-term follow-up of this patient and the evaluation of the efficacy of ensartinib will be continued. Furthermore, we will also pay close attention to whether the patient develops ensartinib resistance in the future.

## Data Availability

Not applicable.

## References

[1] Shaw AT, Gandhi L, Gadgeel S (2016). Alectinib in ALK-positive, crizotinib-resistant, non-small-cell lung cancer: a single-group, multicentre, phase 2 trial. Lancet Oncol.

[2] Gainor JF, Dardaei L, Yoda S (2016). Molecular mechanisms of resistance to first- and second-generation ALK inhibitors in ALK-rearranged lung cancer. Cancer Discov.

[3] Horn L, Wang Z, Wu G (2021). Ensartinib vs crizotinib for patients with anaplastic lymphoma kinase-positive non-small cell lung cancer: a randomized clinical trial. JAMA Oncol.

[4] Shaw AT, Kim DW, Mehra R (2014). Ceritinib in ALK-rearranged non-small-cell lung cancer. N Engl J Med.

[5] Yang Y, Zhou J, Zhou J (2020). Efficacy, safety, and biomarker analysis of ensartinib in crizotinib-resistant, ALK-positive non-small-cell lung cancer: a multicentre, phase 2 trial. Lancet Respir Med.

[6] Horn L, Infante JR, Reckamp KL (2018). Ensartinib (X-396) in ALK-positive non-small cell lung cancer: results from a first-in-human phase I/II, multicenter study. Clin Cancer Res.

[7] Lovly CM, Heuckmann JM, de Stanchina E (2011). Insights into ALK-driven cancers revealed through development of novel ALK tyrosine kinase inhibitors. Cancer Res.

[8] Horn L, Whisenant JG, Wakelee H (2019). Monitoring therapeutic response and resistance: analysis of circulating tumor DNA in patients with ALK+ lung cancer. J Thorac Oncol.

